# A Spiderless Arachnophobia Therapy: Comparison between Placebo and Treatment Groups and Six-Month Follow-Up Study

**DOI:** 10.1155/2007/10241

**Published:** 2007-07-09

**Authors:** Laura Carmilo Granado, Ronald Ranvaud, Javier Ropero Peláez

**Affiliations:** ^1^Institute of Psychology, University of São Paulo, 1721 Avenue of Professor Mello Moraes, 05508-030 São Paulo, Brazil; ^2^Department of Physiology and Biophysics, Institute of Biomedical Sciences, University of São Paulo, 1524 Avenue Professor Lineu Prestes, Prédio Biomédicas I Cidade Universitária, 05508-900 São Paulo, Brazil; ^3^Department of Electronic Systems Engineering, Polytechnic School, University of São Paulo, 802 Rua Alameda Barros, Apartment T3, 01232-000 São Paulo, Brazil

## Abstract

We describe a new arachnophobia therapy that is specially suited for those individuals with severe arachnophobia who are reluctant to undergo direct or even virtual exposure treatments. In this therapy, patients attend a computer presentation of images that, while not being spiders, have a subset of the characteristics of spiders. The Atomium of Brussels is an example of such an image. The treatment group (*n* = 13) exhibited a significant improvement (time × group interaction: *P* = .0026) when compared to the placebo group (*n* = 12) in a repeated measures multivariate ANOVA. A *k*-means clustering algorithm revealed that, after 4 weeks of treatment, 42% of the patients moved from the arachnophobic to the nonarachnophobic cluster. Six months after concluding the treatment, a follow-up study showed a substantial consolidation of the recovery process where 92% of the arachnophobic patients moved to the nonarachnophobic cluster.

## 1. INTRODUCTION

According to the DSM-IV manual (American Psychiatric Association [1]), specific phobias are anxiety disorders that are characterized by an excessive, unreasonable, and persistent fear that is manifested by the presence or expectation of an object or feared situation (phobic situation). The manual states that 9% of the population suffers from specific phobias.

Spider phobia is one of the most common specific phobias (Bourdon et al. [2]). Arachnophobic individuals develop an avoidance behavior for all contexts related to the animal (APA [1]). Many patients are so afraid of being confronted by the phobic object that they refuse to undergo any kind of therapy (Marks [3]).

Existing therapies range from those that confront the patient with the real spider, such as “in vivo” exposure therapy (Ost [4]), to those that avoid this confrontation by requiring the patient to imagine situations involving spiders (Hecker [5]). In between, several therapies try to minimize the anxiety of the direct exposure by using computer simulations in which either the patient himself (Garcia-Palacios et al. [6, 7]) or a “virtual” person guided by the patient (Gilroy et al. [8, 9]) interacts with a “virtual” spider.

The treatment proposed here (SLAT: spiderless arachnophobia therapy) does not use any spider, neither real nor virtual or imaginary. It is specifically oriented to those patients with severe arachnophobia that would not undergo any kind of therapy involving a spider. This treatment makes use of the idea that aversive information does not need to be perceived consciously to trigger an emotional response. Nonconscious processing mechanisms of emotionally relevant stimuli are sufficient to activate the autonomic components of a phobic reaction (Öhman and Soares [10, 11]). From the neural point of view, fearful information does not need to reach cortical levels to generate the typical fear response. Individuals with bilateral destruction of the visual cortices exhibit amygdala responses to emotional faces even when brain damage is recent so that cortical networks have had too short time to reorganize (Pegna et al. [12]). In this case, the amygdala activation requires mediation by thalamic (pulvinar nucleus) or tectal (superior colliculus) areas (Morris et al. [13]; Pegna et al. [12]).

The thalamus and amygdala are, according to LeDoux et al., responsible for recognizing fearful stimuli and triggering subsequent autonomic responses such as increased heart rate, respiration, and sweating (LeDoux [14]; Doyére et al. [15]). According to these authors, when an aversive stimulus arrives at the thalamus, it passes rough, almost archetypal information, directly to the amygdala, producing a rapid response to the possible danger.

**Figure 1:**
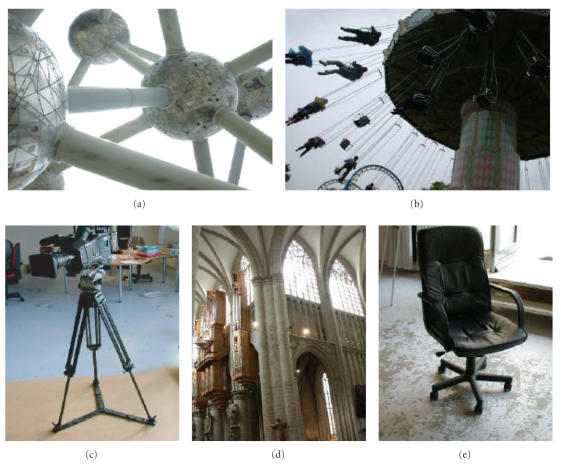
Some “SLAT” images used in the treatment.

The therapy proposed in this paper makes use of these ideas by presenting to the patient a collection of images that contain a reduced subset of the features of a spider. Figure 1 shows some of these images: the Atomium of Brussels in which the spheres resembles the spider's body, a carousel in which the seats hang like the preys of a spider, a tripod whose legs are articulated like spider's legs, and so forth. These images, sharing a limited subset of features of a spider, were called SLAT images. After a preliminary presentation, only the images in which the features of the spider appear in a subtler way are kept in the final presentation. The images that evoke spider-related feelings above a certain degree are discarded from the final therapeutic set (see Section 2.3.2). To avoid the patient's thoughts related to spiders while seeing the treatment presentation, the patient is given a question that should be answered at the end of the run, like “In how many images there is a rounded object?”

## 2. METHODS

### 2.1. Participants

Patients were recruited by means of advertisements in several newspapers and on television. Of the 160 volunteers that made contact with us, 36 with symptoms of severe arachnophobia that were reluctant to undergo other types of treatments were personally interviewed. They were then included in the study if they (1) met DSM-IV criteria of specific phobia (APA [1]) assessed by Structured Clinical Interview for DSM-IV Axis I Disorders (SCID), (2) had been phobic for at least ten years,^1^ (3) did not have any neurological or psychiatric problems, and (4) were classified as arachnophobes according to a *k*-means multivariate analysis.

Four volunteers were excluded because of the three first criteria. A further 6 were excluded because they had difficulty in coming on a regular basis to the university to participate in the experiments.

Regarding the last criterion, the *k*-means multivariate analysis was conducted using as inputs the five measurements obtained from a behavioral avoidance test (BAT) and from the fear of spider questionaire (FSQ); see Section 3. These instruments were applied to the remaining 26 volunteers, and to 29 nonphobic control subjects recruited among the personnel and students of São Paulo University, so that the algorithm could establish two well-defined clusters: the arachnophobic and the nonarachnophobic cluster. After applying the *k*-means multivariate analysis, the 29 control subjects were classified as nonphobic. One of the 26 volunteers was characterized as nonphobic by the *k*-means analysis and was eliminated from the study leaving 25 arachnophobic patients. The mean age and standard deviation of the arachnophobic patients and controls were 31.3 ± 7.4 and 32.6 ± 8.2 years, respectively. The duration of phobia among the patients was 23.0 ± 8.6 years. The five measurements (see the following section) that were used as inputs in the *k*-means algorithm were (a) the distance tolerated to a real tarantula in a BAT; (b) the distance tolerated to a photo of a tarantula in a BAT; (c) the subjective percentage of anxiety according to the subjective units of disconfort scale (SUDS), using a real tarantula; (d) the percentage of anxiety with a photo of a spider; (e) the numerical result of the FSQ test.

The chief advantage of the *k*-means algorithm is that it uses a multivariate approach (here, 5 measurements) in order to separate phobic from nonphobic subjects. This procedure is more robust than adopting only one measurement, such as the BAT or the result of the FSQ, as conventionally used for separating phobic from nonphobic subjects. It is also important to remark that the *k*-means algorithm does not use any arbitrary parameter that can bias the results.

### 2.2. Spider phobia assessment techniques

To assess the degree of spider phobia, three different instruments were used. As described, the SCID (First et al. [16]) was used to produce a preliminary selection of participants. Afterwards, the BAT and the FSQ provided the 5 measurements used to evaluate if participants showed improvement.

### 2.2.1. Structured Clinical Interview for DSM IV Axis I Disoders (SCID)

To verify that patients met DSM-IV criteria for specific phobias (300.29), all of them underwent an SCID (First et al. [16]).

### 2.2.2. Behavioral assessment test (BAT)

The BAT is a widely used measurement of clinical improvement in specific phobias (Lang and Lazovick [17]; Lang et al. [18]). It consists of an artificial situation in which the subject approaches the phobic object until discomfort sets in. The experimenter measures the distance from the subject to the object and assesses the subject's anxiety level using, in our case, the SUDS scale (Wolpe [19]). These tests usually start at 5 meters from the real spider, but in this study the initial distance was established as 25 meters because of the severity of arachnophobia in our patients.

The BAT was performed in two stages: first with a photo of a tarantula (Grammostola acteon, 20 cm) and afterwards with a real tarantula. In both cases the phobic object was placed at the end of a 25-meter long corridor. Before beginning the test, an assistant read the instructions to the subject: “This is a behavioral assessment test and is not part of the therapy. You are free to refuse my suggestions. Walk the farthest you are able to approximate to the spider at the end of the corridor without forcing yourself. I will remain at this point until you stop.” When the subject stops less than one meter from the object, the assistant says: “Touch the photo” or “Touch the cage” in the case of the real tarantula.

Note that instead of asking the patient to approach as much as possible to the spider, the patient is asked to approach to the spider as much as possible without forcing himself. This kind of suggestion guaranteed complying with the desire of patients of not confronting in any way the phobic object.

The BAT was rated by measuring the distance from the subject to the phobic object, starting at 25 meters. The BAT score ranged from 26 if the subject refused to do the test, to −1, if the subject opened the lid of the cage. When subjects stopped, the assistant applies the SUDS by saying: “Please, rate you anxiety from 0% to 100%, 100% being the greatest fear you have had in your life.”

### 2.2.3. Fear of spider questionnaire (FSQ)

The fear of spiders questionnaire (FSQ) assesses the subjective perception of spider fear (Szymanski and O'Donohue [20]). It is composed of 18 questions rated on a 1–7 Likert scale (1 = I strongly disagree, 7 = I strongly agree). The FSQ was able to discriminate between phobics and nonphobics, F(1.111) = 5.99, *P* < .01, F(1.76) = 13.28, *P* < .01, respectively (Szymanski and O'Donohue [20]). It also provided evidence for the improvement of phobic patients following a cognitive restructuring treatment (comparing pretest to posttest: *t*(37) = 4.38, *P* < .01, *t*(79) = 5.09, *P* < .01, resp.). When applied to nontreated subjects, the instrument did not show improvement from pretest to posttest. This instrument has an internal consistency of 0.92 with a split half reliability of 0.89.

### 2.3. Presentation of “SLAT” figures

The presentation used in the SLAT consists of an initial set of 165 images, 124 of them having some features that resemble any of the characteristics (color, shape, texture, etc.) of a spider and were selected as explained in Section 2.3.1. Examples include the image of a person with a Rastafarian hair style, the Atomium of Brussels, a carousel, and so forth.

The remaining 41 images were neutral and were selected with the purpose of making it more difficult for the subject to realize there were SLAT images in the presentation.

The placebo group presentation consisted of a sequence of images without arachniform features. Among the selected figures, there were abstract or surreal paintings that might induce placebo subjects to think there was something hidden in the figures.

### 2.3.1. Selection of figures

The images were selected from the Internet. We chose 132 images with spider features and 44 neutral images. The features that were selected in the images were related, for example, to the radial symmetry of spiders, the design of their webs, their texture, the way they articulate their legs, the hook-like shape of their extremities, or the fact that they hang from a string.

For validating our selection, 43 nonarachnophobic persons were asked to rate, on a 0 to 10 scale, the content of spider features in all the images. Not to bias the process of rating the images, no instructions related to what features to consider in rating the images were given to these persons.

It was necessary to establish a threshold in this scale for separating SLAT images from neutral images. This threshold was obtained by means of the Bayes decision rule that yields a threshold of 0.92. Images with a greater rate were classified as SLAT images, and images with a lower rate were classified as neutral. According to this rule, 8 of the figures initially classified as SLAT images were neutral, and 3 neutral figures were SLAT images. Therefore, a total of 11 images were excluded from the final therapeutic repertoire. To apply the Bayes decision rule, a histogram was created giving the probability of finding a SLAT image inside intervals of 0.6 unit length in the 0 to 10 “arachniform scale.” The same was done with neutral images. We replaced both histograms by two curves after smoothing the histograms by using interpolation by splines. The intersection of the two curves yielded the value of 0.92 that served to discriminate between SLAT and neutral images.

### 2.3.2. Adjustment of presentation intervals

One of the assumptions that served to delineate the SLAT (see assumption (a) in Section 4.1) deals with avoiding a high activation in the neural circuits involved in fear. For this reason, we elaborated a procedure to exclude from the final therapeutic presentation those images that might produce discomfort in the patients, keeping only the more comfortable images that would probably not produce a high degree of activation in these neural circuits.

We adopted the following procedure.

(a) Once the entire set of figures had been shown to the patient in a preparatory presentation, we asked the patient to see the figures once more and collaborate with us to determine the adjusted duration, *T*_ad_, of each one of the images. The patient was instructed as follows: “Each one of the following images will be presented by default for 5 seconds. If you do not like the image, press the “Enter” button to pass to the following image sooner. The sooner you press the button, the more fearful we will understand the image to be for you.”

(b) After seeing all images the subjects were asked:

1. Which images, if any, are intolerable?
2. Which images are tolerable?
3. Which images are so nice that you might place them in your bedroom?

With all this information, nine rules were applied to obtain the final duration of each image, *T*_ad_, in the presentation. As some patients were faster than others in pressing the “Enter” button, the average time *T*_m_ for each subject served as the patient's unit of time.

In the following rules, times *T*_0_, *T*_1_, and so on were set as arbitrary multiples of *T*_m_. The adjusted duration of each image, *T*_ad_, was obtained by multiplying the duration chosen by the subject in the preparatory presentation, *T*, by a coefficient calculated as follows.

We defined three thresholds: *T*_0_ = *T*_m_/5, *T*_1_ = *T*_m_/2, *T*_2_ = *T*_m_/3.

1. If *T* < *T*_0_, the image was eliminated from the presentation.
2. Intolerable images with *T* < *T*_1_ were also eliminated.
3. *T*_ad_ = 0.2 ∗ *T* in intolerable images with *T* > *T*_1_.
4. *T*_ad_ = *T* for tolerable images with *T* < *T*_2_.
5. *T*_ad_ =1.5 ∗ *T* for tolerable images with *T*_1_ > *T* > *T*_2_.
6. *T*_ad_ = 1.8 ∗ *T* for tolerable images with *T* > *T*_1_.
7. *T*_ad_ = 2 ∗ *T* in images deemed nice.
8. Other images, not included in previous groups, maintained their time *T*.
9. To make the total presentation time equal to 12 minutes, each *T*_ad_ was multiplied by 12 and divided by the total duration (in minutes) of the presentation.

All procedures were the same for the placebo group.

### 2.4. Procedure

This research was approved by the Ethics Committee on Research of the Institute of Psychology of the University São Paulo.

As mentioned in Section 2.1, of the 160 patients that contacted us, 36 were interviewed and 25 were included in the experiment. These patients signed forms, agreeing to participate in either the placebo or treatment group, and allow the use of collected data for research. Patients were randomly divided into two groups: treatment (*n* = 13) and placebo (*n* = 12).

After adjusting the timing of the presentation, a personalized CD was prepared for each patient. In the following session, this CD was given to the patient. The patient was then instructed to run the presentation twice a day at home preferably during moments in which she/he was not tired or under stress. Prior to each presentation run, the patient was given one question to answer at the end of the run. These questions were intended to distract the patient from arachniform features in the images. Examples include: “In how many images there is an animal?” or “In how many images there is a rounded object?” When answering the question, the patient was instructed to write, beside the answer, the date and time she/he ran the presentation. Every week these data were checked out in order to verify the rate of cooperation of patients and to encourage noncooperative patients, if any. In all subjects, the cooperation was satisfactory and no statistics were deemed necessary to measure the rate of cooperation.

To assess progress during the treatment, placebo and treatment subjects underwent the BAT (including the SUDS) each week. In the last week, the FSQ was also applied. Experiments were carried out in three stages. In stage 1, data collected during these first four weeks were used to compare placebo and treatment groups. A period of four weeks was established prior to the experiment with the intention of minimizing the duration of the experiment in order to avoid drop out. In stage 2, the treatment group (but not the placebo) was asked (and luckily agreed) to continue for two more weeks to assess if this additional time might help the treated group to achieve a more substantial recovery. They were evaluated at the end of the 6th week.

In stage 3, after the fourth week, placebo subjects were invited to receive the SLAT. The ten subjects that were accepted were treated for 6 weeks and evaluated after the 4th and 6th weeks.

## 3. RESULTS

### 3.1. Comparison between placebo and control groups at the beginning of the study

There were no difference between the placebo (*n* = 12) and treatment (*n* = 13) groups at the beginning of the study in the following demographic and clinical variables: age, F(1,23) = 0.3315, *P* = 0.5703; duration of phobia, F(1,23) = 3.8758, *P* = .0611. No significant differences were found in behavioral variables during the initial BAT test with the real spider BAT: F(1,23) = 0.0015, *P* = .9692; SUDS, F(1,23) = 0.0739, *P* = .7881; or with the spider photo BAT, F(1,23) = 1.6764, *P* = .2082; SUDS, F(1,23) = 0.0003, *P* = .9866. No significant difference was found in the subjective measure of fear of spiders, FSQ: F(1,23) = 0.020, *P* = .8895.

Of the 13 treatment subjects, 3 refused to stay at any distance from the real spider if the spider was visible. They received an arbitrary score of 26, one meter more than the maximum score of 25 meters used in the BAT test. Regarding the test with the spider photo, one subject refused to stay at any distance in which he could see the photo. Analogously, we assigned a score of 26 meters in the BAT test to this subject. We emphasize that, different from previous studies in which the initial distance of the BAT test was standardized to 5 meters, this distance was augmented to 25 meters because of the desire of the patients not to confront the spider in anyway.

### 3.2. Comparative evolution of placebo and treated groups

**Figure 2:**
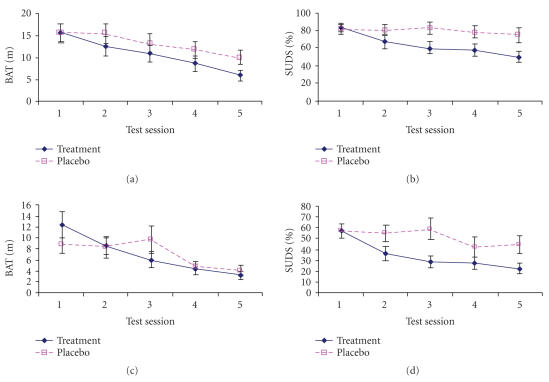
Time course of the BAT and SUDS means with a real spider, (a) and (b), and with a spider photo, (c) and (d), for placebo and treatment groups. Vertical segments indicate standard error.

Table 1 shows the mean and standard deviation (in parenthesis) of the various groups evaluated. The percentage improvement (Table 2) was calculated by dividing the absolute improvement in each measure by the initial measure. After 4 weeks, the percentage improvement in all measurements was higher in the treated than in the placebo group. During the presentation of the real spider, the percentage improvement in the BAT was more than twice as high (61.6% versus 28.8%) in the treated than in the placebo group (see Table 2). The SUDS was more than six-fold (40.3% versus 5.9%) higher. The same measurements made with the spider photo yielded a percentage improvement of 19.3% (66.6%–47.3%) in the BAT and 32% (53%–21%) in the SUDS. Differences between placebo and treated groups were consistent throughout the four weeks of the experimental procedure (see evolution of measures in Figure 2).

Improvement in the FSQ was 13.1% (28.8%–15.7%) higher in the treatment than in the placebo group.

### 3.2.1. Repeated measures multivariate ANOVA

A 2 (group) × 5 (times) repeated measure multivariate ANOVA (Hair et al. [21]) was conducted to evaluate whether the differences between placebo and treated groups were significant. In this multivariate analysis, 4 simultaneous variables were used: BAT and SUDS for real spiders; and BAT and SUDS for spider photo. By analyzing the results of the multivariate ANOVA, we conclude that the significant time effect F(4,92) = 14.5475, *P* < .0001, and the significant group effect F(1,23) = 4.5678, *P* = .04344 show the effectiveness of the treatment. The significantly different time-course of the improvement in the two groups is also reflected in a significant group × time effect F(4,92) = 4.4217, *P* = .0026. In order to evaluate how the test with the real spider and the test with the spider photo contribute to these results, a 2-group, ×5 times, multivariate ANOVA was performed, first with the BAT and SUDS of the real spider and then with the BAT and SUDS of the spider photo. The test with the real spider yielded a significant group × time interaction: F(1,23) = 7.981610, *P* = .009598, MS = 1369.772 while the test with the spider photo yielded a moderate group × time interaction F(1,23) = 2.908077, *P* = .101608, MS = 750.1708. The FSQ also yielded a nonsignificant 2 (groups) ×2 (time = pretreatment versus post treatment) interaction F(1,23) = 1.833, *P* = .188. The difference between BAT and SUDS tests and the FSQ test results are analyzed in the discussion.

### 3.3. Results of prolonging treatment until the sixth week

After the four weeks in which placebo and treated subjects were compared, treated subjects continued receiving the SLAT for two more weeks, achieving 76.6% improvement in the BAT and 45.6% in the SUDS with the real spider. With the spider photo, there was an 88.5% improvement in the BAT; a 61.4% improvement in the SUDS, and a 40% improvement in the FSQ.

The results of the treated placebo were consistent with the results of the treatment group (see Tables 1 and 2).

### 3.4. Six-month follow-up study

A six-month follow-up study was also performed. It showed a substantial consolidation of previously obtained results. There was 90.2% improvement in the treatment group in the BAT test: patients were capable of approaching a live tarantula at 2(3.9) meters (on average), six patients opened the lid of the tarantula cage and, of these, three patients touched the tarantula (*Grammostola acteon*, 14 cm, the initial one died).

In the case of the follow-up study with the treated placebo patients, there was an improvement of 79.2% in the BAT test. Three of them opened the lid of the cage and two of them touched the tarantula.

Only one patient dropped out of the follow-up study.

### 3.5. *k*-means cluster analysis

A *k*-means multivariate cluster analysis was used to assess the number of patients that made the transition from arachnophobic to normal during treatment. Five variables were used to characterize each subject: BAT and SUDS with real spider, BAT and SUDS with photo of a spider, and FSQ. The algorithm was applied with these five variables gathered from the 25 arachnophobes at the beginning of treatment, and from 29 normal subjects recruited in the university. The *k*-means algorithm was initially used to eliminate nonphobic subjects from the group of volunteers, as explained in Section 2.1. To calculate the percentages of patients that migrated from arachnophobic to normal along the different stages of the experimental procedure (see Table 2), the *k*-means algorithm was fed with the scores of the participants in each one of the stages (BAT spider, BAT photo, SUDS spider, SUDS photo, and FSQ).

During the four weeks of treatment, 41.7% of individuals in the treatment group and 25% of the placebo group moved over to the normal condition. When the placebo group was treated, 50% fell in the normal group.

A more substantial improvement was evident in the follow-up, six months after the conclusion of treatment: 91.7% of individuals in the treatment group and 90% of the treated placebo group were classified as nonarachnophobes. These results are discussed below.

## 4. DISCUSSION

In this section, the following topics will be discussed:

1. the hypothetical assumptions taken into consideration to elaborate the therapy;
2. the neurocomputational background of the therapy;
3. the influence of the BAT assessment test in the efficacy of SLAT;
4. the delay of improvement in the FSQ;
5. the therapeutical limitations of the procedure;
6. suggestions for further studies.

### 4.1. Hypothetical assumptions for elaborating the SLAT

Two hypothetical assumptions that are consistent with neurological findings served to delineate the methodology of SLAT. The results of the therapy, however, are not intended to assess the validity of these preliminary assumptions, which would require much further confirmation.

 (a) The first assumption is that some connections from thalamus to amygdala are abnormally potentiated in phobic patients, possibly because of a process in which a conditioned stimulus (CS), the phobic object, is associated with an unconditioned stimulus (US) such as a loud sound or an acute pain. The possibility of plastic changes taking place in the thalamo-amygdala pathway is supported by the work of Doyére et al. [15], in which they were able to induce long-term potentiation (LTP) in thalamic and cortical inputs to the amygdala in freely moving rats, demonstrating that LTP in thalamic inputs is much more persistent and long-lasting than LTP in cortical inputs. LeDoux, Schafe et al. (Apergis-Schoute et al. [22]) have further shown that intralaminar thalamic neurons contribute to presynaptic plasticity in the thalamo-amigdaloid pathway during fear conditioning. Thalamic intralaminar neurons are also described as a locus of functional CS-US convergence for fear conditioning to acoustic stimuli (Cruikshank et al. [23]). The possibility of altering these circuits by means of either habituation to the spider or by cognitive-behavioral therapy is also mentioned, for example, by Veltman et al. [24] and Paquette et al. [25].

**Figure 3:**
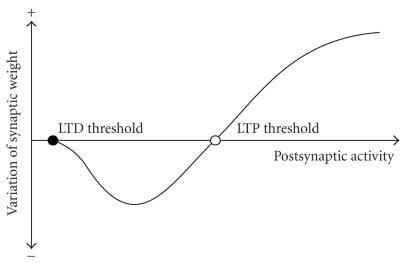
Variation of synaptic efficiency (synaptic weight) in terms of postsynaptic activity. For levels of postsynaptic activity above the LTP threshold, synaptic potentiation (positive variation of synaptic weight) takes place. Between the LTD and LTP thresholds, synaptic depression (a negative variation of synaptic weight) occurs. Below the LTD threshold there is no variation of synaptic efficiency.

Regarding the degree to which plastic changes would take place in the thalamo-amygdaloid pathway, it is worth mentioning that postsynaptic voltage value is critical to determining whether a synapse is reinforced or depressed (Figure 3). According to Figure 3, postsynaptic depolarization determines the potentiation or depression of a given synapse. If the value of postsynaptic depolarization is greater than a threshold, called the LTP threshold, active synapses are potentiated (i.e., increment their synaptic connectivity or synaptic weight); below this threshold they are depressed (Artola and Singer [26]; Bear et al. [27]) (these synapses experiment a decrement of their synaptic connectivity or synaptic weight). If the postsynaptic depolarization is very low, synaptic depression is small or null.

We conjectured that the effectiveness of SLAT depends on activating neurons that project from thalamus to amygdala in such a way that they are inside the depression interval. Unfortunately, depression intervals vary for each synapse according to a synaptic property called metaplasticity. The same postsynaptic activity may produce potentiation in one synapse and depression in another while leaving a third unaltered. We were also unable to directly evaluate the postsynaptic activity that a given SLAT figure produced in these neurons.

Despite all these difficulties, we conjectured that the fear reaction produced by SLAT figures was correlated to the postsynaptic activity in neurons in the thalamo-amygdaloid pathway. To avoid potentiation and favor depression, fearful images were omitted from the presentation (see Section 2.3.2). The duration of the remaining images were adjusted so that comfortable images were exhibited during a longer time and less comfortable images during a shorter interval.

(b) The second hypothetical assumption that served to delineate SLAT is related to the nature of the archetypal information that, according to LeDoux, is relayed from the thalamus to the amygdala. Morris et al. [28] found that the amygdala appears to sum, in a nonlinear manner, individual responses to specific facial features. A two-stage theory for facial perception of emotions was proposed by De Bonis et al. [29] and tested by Morris et al. [28], who concluded that “the perception of emotional expressions depends on an initial processing of individual facial features followed by a nonlinear association of the different components.” According to Weinberger and collaborators (Lennart and Weinberger [30]; Edeline and Weinberger [31]), the thalamus is able to recognize features, augmenting its response to a specific feature that was previously paired to a US.

### 4.2. Neurocomputational foundations

**Figure 4:**
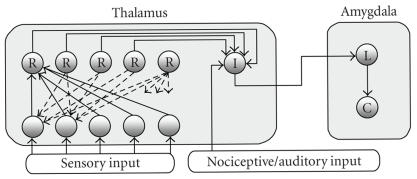
Hypothetical arrangement of thalamus and amygdala connections, used in the computational model that inspired the therapy here described (SLAT). R: thalamic reticular neurons; I: thalamic intralaminar neurons; L: lateral nucleus of the amygdale; C: central nucleus of the amygdala. Due to a competitive process performed between reticular neurons in the model, each one of them responds to a specific feature of a sensory pattern (Peláez [32, 33]). A similar competitive process takes place between intralaminar neurons, each one responding to a specific combination of features. Therefore, a certain number of features, that is, reticular neurons, are necessary for firing a specific intralaminar neuron. When this number is low, a low postsynaptic activity in intralaminar neuron favors synaptic depression, according to Figure 3, thereby reducing the possibility of future intralaminar neuron firing. In this way, the thalamic-amygdala pathway is depressed in the computational model.

Neurocomputational models (Peláez [32, 33]) are consistent with the two-stage theory, conjecturing that the first stage of the process, the preliminary processing of individual features, is performed in the thalamus. According to these models, in the thalamus each sensory pattern is represented as a vector with components in a coordinate frame in which each axis corresponds to a specific feature of the pattern. Each one of these axes/features corresponds to the output of a thalamic reticular neuron. The output of these reticular neurons (Crabtree and Isaac [34]) is nonlinearly summed by intralaminar neurons (see Figure 4) and if this sum exceeds a threshold, the result is relayed to the amygdala. According to the computational model, the set of axes/features created by the firing of reticular neurons in the thalamus, constitute a code that identifies, in a rough way, each input pattern. This code would correspond to the rough, almost archetypal description of the aversive stimuli, that, according to LeDoux and colleagues (LeDoux [14]; Doyére et al. [15]), is passed from the thalamus to the amygdala.

According to the first assumption, a way of depressing thalamo-amygdaloid synapses would be by avoiding high post-synaptic potentials in thalamo-amygdaloid neurons by means of reducing the intensity of phobic stimuli (Figure 3). A possible way of reducing this intensity would be by masking or obscuring the phobic object. However, a masked or obscured phobic object is still intense enough to fire the amygdala (Whalen et al. [35]) and aversive for patients.

Instead of reducing the duration or intensity of spider images, we propose to reduce the number of arachnoid features present in each image. According to the second assumption, when the number of arachniform features in the input pattern is reduced, the activation of intralaminar neurons (computing the sum of these features) is also reduced. This lower activation of intralaminar neurons contributes to reduce the activation of the neurons in the thalamo-amigdaloid axis, so that their synapses would undergo depression instead of potentiation. Therefore, when, instead of the spider code, a code with a smaller repertoire of arachniform features is relayed, neurons in the thalamo-amigdaloid pathway are hypothetically less activated, their synapses more prompted to undergo depression rather than potentiation.

### 4.3. Influence of the BAT assessment test in the efficacy of the SLAT

Both treatment and placebo groups underwent BAT and SUDS assessment test weekly. Volunteers were told to approach the spider without forcing themselves. The purpose of this instruction was to adhere, during the BAT and SUDS tests, to the principles that inspired the therapy, that is, to avoid any stimuli that could contribute to enhance thalamo-amygdala connectivity.

It could be argued that the BAT assessment test could, by itself, have a therapeutical effect over arachnophobia. This effect might be thought to be responsible for the improvement observed in the placebo group. However, as shown in Section 3.2, improvement of patients in the treatment group was significantly better than that of patients in the placebo group.

### 4.4. The delay of improvement in the FSQ

Many patients reported that they did not realize that they had lost their fear of spiders until they were confronted to a real spider during their daily life. They had the strange sensation of not reacting with fear when, for the first time after treatment, they saw a real spider. Since during daily life, a real confrontation with a spider is an unpredictable event, the realization of having lost the fear varies from individual to individual. The BAT assessment test, independently of its possible placebo effect, could contribute to accelerate this process of realization.

Related to this, we observed that the improvement in the FSQ was delayed in comparison to the improvement in the automatic responses measured by the BAT and SUDS. This is consistent with the reasonable supposition that patients did not realize that they had lost their fear until they actually confronted a real spider during their daily life situations. Depending on the frequency with which they actually confronted a spider in their daily lives, the realization of recovery took a shorter or longer time in the different patients. This fact was reflected in the follow-up study that was carried out six months after the conclusion of the treatment.

### 4.5. Therapeutical limitations

Although the 25 subjects that took part in the experiment came from a very large sample of 160 arachnophobic volunteers, there were no volunteers above the age of 46. Taking into account that neural plasticity depends on age (Burke and Barnes [36]) and that our experiments were not able to assess the therapeutic effect of SLAT in elderly people, we suggest to apply the SLAT to patients below the age of 46, until performing an assessment with older volunteers in the future.

### 4.6. Suggestions for further studies

The 160 arachnophobic patients that contacted us were classified in terms of their degree of arachnophobia. Among the six with the highest scores, three of them suffered thyroid hormone impairment. We wondered whether this coincidence might be a possible psycho-somatic effect produced in the long run by arachnophobia. A similar case of thyroid hormone alteration was found in the literature (Friedman et al. [37] ) among women with posttraumatic stress disorders. These considerations motivate a study to assess the relationship between thyroid hormone alteration and phobias.

According to our theoretical assumptions, the SLAT acts at subcortical levels. Neuroimaging studies could help to evaluate this assumption by comparing the brain activation before and after the SLAT. A similar comparison was done by Paquette et al. [25], in which arachnophobic patients were treated with cognitive behavioral therapy. This study concluded that the dorsolateral prefrontal cortex and the parahippocampal gyrus diminished their activation significantly after treatment with cognitive behavioral therapy. In the case of the SLAT, we expect that reduction of activity in the dorsolateral prefrontal cortex and the parahippocampal gyrus will be preceded by reduced activity of amygdala and superior colliculus. This sequence would be consistent with the fact that during the SLAT, improvement in the BAT test (measuring automatic responses) proceeded the improvement in the FSQ tests (measuring cognitive variables related to fear of spiders).

## 5. CONCLUSION

A novel technique for treating spider phobia, that does not require any use of spiders, was described and tested. In the SLAT, here described, each patient is given a personalized presentation in a compact disk, containing a set of images that, although not containing spiders, present subsets of spider characteristics. The degree to which each image evokes a spider in different patients is different. The most evocative images are excluded from the personalized presentation whereas the less evocative images are presented to the patient during a longer interval (see Section 2.3.2). Regarding the subtlety of the images, two treatment group patients declared that they thought they were in the placebo group because their presentation caused no discomfort at all.

To compare the evolution of the placebo and treatment groups, a four-week experiment was designed. Treatment and placebo groups went through their corresponding presentation twice a day and came once a week to the university to apply the BAT and SUDS tests. To carry out these tests, instead of encouraging the subjects to approach as much as possible to a spider, they were told to approach the spider, but without forcing themselves. They could also refuse to do the test, which was the case of three treatment subjects in their initial evaluation (see Section 3.1). This kind of suggestion respects the desire of the subjects of not confronting the spider in any way, and is coherent with the main philosophy of the procedure, according to which the subtler the better. The improvement in every measure of phobia was higher for the treatment group than in the placebo group (see Tables 1 and 2). Moreover, the repeated measures multivariate ANOVA showed that the patients' improvement was not due to a placebo effect (group × time interaction: F(1,23) = 7.98, *P* = .0096).

In the follow-up study performed after six months, 91.7% of the patients in the treatment group were classified as nonarachnophobes by the *k*-means algorithm, six patients of this group opened the lid of the tarantula cage, and, of these, three touched the tarantula.

The therapy proposed here was aimed at subconscious, automatic responses, while behavioral or psychoanalytic therapies emphasize the rational control of fear reactions. According to LeDoux [38], the alteration of fear behavior can be produced by the cortical control of fear reactions without the actual deletion of what LeDoux calls “fear memories,” that once established become relatively permanent. These “fear memories” were intentionally the targets of the therapy proposed in this paper.

SLAT is particularly appropriate for, but not exclusive to, those patients who, because of the severity of their arachnophobia or whatever other reason, are unwilling to undergo therapies that involve any real, imagined or virtual spider. The theoretical basis of the therapeutic strategy was aiming to produce plastic changes in the thalamo-amygdaloid circuit responsible for the subconscious, automatic reactions triggered when the subject sees a spider. The therapy might have been effective for other, fortuitous, reasons, but the consistency with the theoretical basis that motivated it (Sections 4.1 and 4.2) is very encouraging, both from a practical point of view, providing an additional strategy to deal with certain phobias, and from a theoretical point of view, motivating further studies to test these ideas.

## Figures and Tables

**Table 1:** Means and standard deviations (in parenthesis) of the BAT, SUDS, and FSQ scores. Treatment (*n* = 13) and placebo (*n* = 12) group scores were gathered and compared at the end of the 4th week. Treatment group continued treatment until the 6th week. After 4 weeks, ten placebo subjects also underwent treatment, and their improvement was calculated at the 4th and 6th weeks of treatment. Six months later, a follow-up study was performed.

	Real spider		Spider photo		FSQ
	BAT	SUD	BAT	SUD		

**Treatment**					
Start	15.6 (7,7)	82.8 (17,9)	12.3 (8.6)	56.9 (24.3)	105.5 (11.2)
4 weeks	5.9 (4.4)	50 (22.4)	3.2 (2.7)	22.3 (17)	74.7 (23.2)
6 weeks	3.9 (5.4)	43.5 (32.5)	1.4 (2)	17.7 (19.3)	63 (30.2)
6 months (follow-up)	2.01 (3.9)	32.1 (27.5)	1.0 (1.53)	14.6 (19.1)	48.2 (27.0)

**Placebo**					
Start	15.7 (7.2)	80.8 (19.2)	8.7 (4.8)	57.1 (22.8)	107.7 (16.8)
4 weeks	10 (5.2)	73.8 (25.9)	4 (3.7)	44.2 (28.7)	90.8 (22.7)

**Treated placebo**					
Start	10.8 (5.3)	81 (20.9)	4.6 (3.6)	49 (28.8)	99.1 (15.5)
4 weeks	5.9 (5.2)	60.5 (26.5)	2.1 (2.5)	27.9 (31)	73.4 (23.1)
6 weeks	3.1 (4.9)	45.6 (33.9)	1.1 (1.7)	23.2 (29.2)	59.6 (26.4)
6 months (follow-up)	1.8 (3.00)	34.2 (27.2)	0.6 (1.1)	19.9 (21.0)	49.2 (28.4)

**Treatment and treated placebo**					
Start	13.5 (7.0)	82.0 (18.8)	9.0 (7.8)	53.5 (26.0)	102.7 (13.3)
4 weeks	5.9 (4.6)	54.6 (24.3)	2.8 (2.6)	24.7 (23.6)	74.1 (22.6)
6 weeks	3.6 (5.1)	44.4 (32.4)	1.3 (1.7)	20.1 (23.7)	61.5 (28.0)
6 months (follow-up)	1.91 (3.4)	33.1 (26.7)	0.8 (1.3)	17 (19.2)	48.6 (26.9)

**Table 2:** Improvement of the BAT, SUDS, and FSQ scores in Table 1 expressed in percentages. The percentage of improvement was calculated from Table 1 by dividing the measurement by the initial score. The last column exhibits the percentage of patients that migrated to the condition of normal subjects, according to the *k*-means algorithm. According to this, in six months, 91.7% of the treatment-group subjects became nonarachnophobes.

	Real spider		Spider photo		FSQ	Recovery (*k*-means) (%)
	BAT	SUD	BAT	SUD				

**Treatment**						
Improv. (%) 4 weeks	61.6 (19.4)	40.3 (22,9)	66.6 (31.2)	53 (51.7)	28.8 (20.5)	41.7
Improv. (%) 6 weeks	76.6 (27.9)	45.6 (46.1)	88.5 (17.1)	61.4 (53.6)	40 (27.1)	50
Improv. (%) (follow-up)	90.22 (25.74)	62.0 (2.7)	87.49 (17.52)	70.6 (37.4)	55.2 (23.4)	91.7

**Placebo**						
Improv. (%) 4 weeks	28.8 (31.8)	5.9 (40.9)	47.3 (37.3)	21 (36.9)	15.7 (18.3)	25

**Treated placebo**						
Improv. (%) 4 weeks	46.8 (31.5)	24.1 (28.4)	46.2 (37.2)	42.3 (42.1)	26.2 (19.1)	50
Improv. (%) 6 weeks	71.2 (38.7)	44.2 (35.1)	67 (40.7)	54 (39)	39.4 (25.8)	50
Improv. (%) (follow-up)	79.2 (33.6)	58.3 (31.1)	87.0 (21.4)	63.3 (40.6)	50.4 (26.7)	90

**Treatment and treated placebo**						
Improv. (%) 4 weeks	55.2 (25.8)	33.3 (26.2)	57.7 (34.7)	48.3 (47.0)	27.7 (19.5)	43
Improv. (%) 6 weeks	74.3 (32.3)	45 (40.8)	79.1 (30.9)	58.1 (46.9)	39.7 (26.0)	50
Improv. (%) (follow-up)	85.2 (29.4)	60.3 (31.3)	87.3–18.9	67.3 (38.1)	53.1 (24.4)	91

## ABBREVIATIONS

ANOVA: Analysis of variance
BAT: Behavioral avoidance test
CS: Conditioned stimulus
DSM IV: Diagnostic and Statistical Manual of Mental Disorderes (4th ed.)
FSQ: Fear of spider questionaire
LTD: Long-term depression
LTP: Long-term potentiation
SCID: Structured Clinical Interview for DSM IV
SLAT: Spiderless arachnophobia therapy
SUDS: Subjective units of disconfort scale
US: Unconditioned stimulus

## Notes

^1^We have arbitrarily chosen this duration as an additional criterion to recruit only severe arachnophobic subjects.

## References

[1] American Psychiatric Association (APA) (1994). *Diagnostic and Statistical Manual of Mental Disorderes*.

[2] Bourdon KH, Boyd JH, Era DS, Burns BJ, Thompson JW, Locke BZ (1988). Gender differences in phobias: results of the ECA community survey. *Journal of Anxiety Disorders*.

[3] Marks IM, Echeburua E (1992). Tratamiento de exposición en la agoraphobia y el pánico. *Avances en el tratamiento psicológico de los trastornos de ansiedad*.

[4] Ost L-G (1989). One-session treatment for specific phobias. *Behaviour Research and Therapy*.

[5] Hecker JE (1990). Emotional processing in the treatment of simple phobia: a comparison of imaginal and in vivo exposure. *Behavioural Psychotherapy*.

[6] Garcia-Palacios A, Hoffman H, Kwong See S, Tsai A, Botella C (2001). Redefining therapeutic success with VR exposure therapy. *CyberPsychology and Behavior*.

[7] Garcia-Palacios A, Hoffman H, Carlin A, Furness TA, Botella C (2002). Virtual reality in the treatment of spider phobia: a controlled study. *Behaviour Research and Therapy*.

[8] Gilroy LJ, Kirkby KC, Daniels BA, Menzies RG, Montgomery IM (2000). Controlled comparison of computer-aided vicarious exposure versus live exposure in the treatment of spider phobia. *Behavior Therapy*.

[9] Gilroy LJ, Kirkby KC, Daniels BA, Menzies RG, Montgomery IM (2003). Long-term follow-up of computer-aided vicarious exposure versus live graded exposure in the treatment of spider phobia. *Behavior Therapy*.

[10] Öhman A, Soares JJF (1993). On the automatic nature of phobic fear: conditioned electrodermal responses to masked fear-relevant stimuli. *Journal of Abnormal Psychology*.

[11] Öhman A, Soares JJF (1994). “Unconscious anxiety”: phobic responses to masked stimuli. *Journal of Abnormal Psychology*.

[12] Pegna AJ, Khateb A, Lazeyras F, Seghier ML (2005). Discriminating emotional faces without primary visual cortices involves the right amygdala. *Nature Neuroscience*.

[13] Morris JS, DeGelder B, Weiskrantz L, Dolan RJ (2001). Differential extrageniculostriate and amygdala responses to presentation of emotional faces in a cortically blind field. *Brain*.

[14] LeDoux JE (1997). Emotion, memory and the brain. *Scientific American*.

[15] Doyère V, Schafe GE, Sigurdsson T, LeDoux JE (2003). Long-term potentiation in freely moving rats reveals asymmetries in thalamic and cortical inputs to the lateral amygdala. *European Journal of Neuroscience*.

[16] First MB, Spitzer RL, Gibbon M, Williams J (1998). Structured clinical interview for DSM IV axis I disorders—patient edition (SCID-I/P, version 2.0. 9/98 revision).

[17] Lang PJ, Lazovick AD (1963). Experimental desensitization of a phobia. *Journal of Abnormal and Social Psychology*.

[18] Lang P, Melamed BG, Hart JA (1970). A psychophysiological analysis of fear modification using an automated desensitization procedure. *Journal of Abnormal Psychology*.

[19] Wolpe J (1973). *The Practice of Behavior Therapy*.

[20] Szymanski J, O'Donohue W (1995). Fear of spiders questionnaire. *Journal of Behavior Therapy and Experimental Psychiatry*.

[21] Hair JF, Anderson RE, Tatham RL, Black WC (1998). Multivariate analysis of variance. *Multivariate Data Analysis*.

[22] Apergis-Schoute AM, Dębiec J, Doyère V, LeDoux JE, Schafe GE (2005). Auditory fear conditioning and long-term potentiation in the lateral amygdala require ERK/MAP kinase signaling in the auditory thalamus: a role for presynaptic plasticity in the fear system. *The Journal of Neuroscience*.

[23] Cruikshank SJ, Edeline J-M, Weinberger NM (1992). Stimulation at a site of auditory-somatosensory convergence in the medial geniculate nucleus is an effective unconditioned stimulus for fear conditioning. *Behavioral Neuroscience*.

[24] Veltman DJ, Tuinebreijer WE, Winkelman D (2004). Neurophysiological correlates of habituation during exposure in spider phobia. *Psychiatry Research*.

[25] Paquette V, Lévesque J, Mensour B (2003). “Change the mind and you change the brain”: effects of cognitive-behavioral therapy on the neural correlates of spider phobia. *NeuroImage*.

[26] Artola A, Singer W (1993). Long-term depression of excitatory synaptic transmission and its relationship to long-term potentiation. *Trends in Neurosciences*.

[27] Bear MF, Connors BW, Paradiso MA (2001). *Neuroscience: Exploring the Brain*.

[28] Morris JS, DeBonis M, Dolan RJ (2002). Human amygdala responses to fearful eyes. *NeuroImage*.

[29] De Bonis M, De Boeck P, Pérez-Díaz F, Nahas M (1999). A two-process theory of facial perception of emotions. *Comptes Rendus de l'Académie des Sciences - Series III - Sciences de la Vie*.

[30] Lennartz RC, Weinberger NM (1992). Frequency-specific receptive field plasticity in the medial geniculate body induced by pavlovian fear conditioning is expressed in the anesthetized brain. *Behavioral Neuroscience*.

[31] Edeline J-M, Weinberger NM (1992). Associative retuning in the thalamic source of input to the amygdala and auditory cortex: receptive field plasticity in the medial division of the medial geniculate body. *Behavioral Neuroscience*.

[32] Peláez JR (1997). Plato's theory of ideas revisited. *Neural Networks*.

[33] Peláez JR (2000). Towards a neural network based therapy for hallucinatory disorders. *Neural Networks*.

[34] Crabtree JW, Isaac JTR (2002). New intrathalamic pathways allowing modality-related and cross-modality switching in the dorsal thalamus. *The Journal of Neuroscience*.

[35] Whalen PJ, Rauch SL, Etcoff NL, McInerney SC, Lee BM, Jenike MA (1998). Masked presentations of emotional facial expressions modulate amygdala activity without explicit knowledge. *The Journal of Neuroscience*.

[36] Burke SN, Barnes CA (2006). Neural plasticity in the ageing brain. *Nature Reviews Neuroscience*.

[37] Friedman MJ, Wang S, Jalowiec JE, McHugo GJ, McDonagh-Coyle A (2005). Thyroid hormone alterations among women with posttraumatic stress disorder due to childhood sexual abuse. *Biological Psychiatry*.

[38] LeDoux JE (1998). *The Emotional Brain: The Mysterious Underpinnings of Emotional Life*.

